# Using objective measures to examine the effect of suspect-filler similarity on eyewitness identification performance - Final Registered Report

**DOI:** 10.1186/s41235-023-00522-w

**Published:** 2023-11-06

**Authors:** Geoffrey L. McKinley, Daniel J. Peterson

**Affiliations:** 1 Minnesota State University, Mankato, USA; 2 Skidmore College, Saratoga Springs, USA

**Keywords:** Lineup construction, Similarity, Multidimensional scaling, Eyewitness memory

## Abstract

When selecting fillers to include in a police lineup, one must consider the level of similarity between the suspect and potential fillers. In order to reduce misidentifications, an innocent suspect should not stand out. Therefore, it is important that the fillers share some degree of similarity. Importantly, increasing suspect–filler similarity too much will render the task too difficult reducing correct identifications of a guilty suspect. Determining how much similarity yields optimal identification performance is the focus of the proposed study. Extant research on lineup construction has provided somewhat mixed results. In part, this is likely due to the subjective nature of similarity, which forces researchers to define similarity in relative terms. In the current study, we manipulate suspect–filler similarity via a multidimensional scaling model constructed using objective facial measurements. In doing so, we test the “propitious heterogeneity” and the diagnostic-feature-detection hypotheses which predict an advantage of lineups with low-similarity fillers in terms of discriminability. We found that filler similarity did not affect discriminability. We discuss limitations and future directions.

**Supplementary Information:**

The online version contains supplementary material available at 10.1186/s41235-023-00522-w.

## Introduction

To date, there have been 397 documented cases litigated by the Innocence Project in which a wrongfully incarcerated individual was later exonerated by DNA evidence (National Registry of Exonerations, 2023). In 261 (66%) of the cases, one or more eyewitnesses erroneously identified the innocent suspect as the perpetrator (National Registry of Exonerations, 2022) rendering it the single greatest contributor for wrongful incarcerations in the USA. Though eyewitness testimony is an indispensable component of the criminal justice system, this underscores what researchers have argued for decades: Eyewitness memory is fallible. Accordingly, it is important that eyewitness memory researchers (a) better understand those circumstances in which witnesses are most likely to make a misidentification, and (b) determine which procedures the criminal justice system can implement to minimize or mitigate the impact of such errors.

These two branches of exploration are commonly referred to as estimator and system variables, respectively (Wells, 1978; however, see Wells, 2020 for a discussion on reflector variables). Estimator variables are factors that influence the likelihood a witness makes an accurate identification, but (critically) are beyond the criminal justice system’s control. For example, one can intuit that the more time a witness has to study a face the more likely they are to make an accurate identification during a subsequent lineup. This intuition is supported by empirical findings (e.g., Palmer et al., 2013), but it is important to note that encoding time is not controlled by the criminal justice system. Accordingly, beyond simply acknowledging that some witnesses will have a poorer or better chance at accurately identifying a suspect by virtue of how much time they had to study the individual’s face, there is little the criminal justice system can do with that information. Conversely, system variables are those variables that affect eyewitness memory that the criminal justice system *can* control. As one might expect, the goal (generally) is to try to institute policies that maximize the likelihood of witnesses making a correct identification while minimizing false identifications. For example, the way in which a lineup is presented to a witness can impact their likelihood of success. Specifically, research suggests that sequential lineups result in worse witness identification performance relative to simultaneous lineups (e.g., Mickes et al., 2012), at least when there is a strict stopping rule (see Horry et al., 2021).

The issue of lineup construction is among the most studied system variables. When an investigator is tasked with constructing a lineup, they are typically provided with (a) a verbal description of the perpetrator (i.e., the individual who actually committed the crime) by the eyewitness and (b) a photograph of the suspect (i.e., the individual identified as someone who could plausibly be the perpetrator). The investigator must then select several photographs of people who are known to be innocent (i.e., fillers) to include in the lineup alongside the suspect. During the filler selection process, it is important to include fillers in such a way that the suspect does not “stand out.” That is, salient, identifiable features such as height and weight should be consistent across the lineup. If, for example, the suspect has a darker complexion than all the fillers, the suspect would unfairly stand out eliciting more identifications of both guilty suspects and, more concerningly, innocent suspects (Wells et al., 1993). Although the potential problems arising from fillers being too different is an intuitive one, researchers have identified that problems can exist along the other end of the continuum as well. Namely, when fillers appear *too* similar to the suspect (imagine the extreme hypothetical in which a suspect’s identical twin is included in the lineup), the task can become too difficult for eyewitnesses and correct identification rates suffer accordingly (Luus & Wells, 1991; see also Fitzgerald et al., 2015). Therefore, the lineup administrator should search for an optimized “sweet spot” of suspect–filler similarity that maximizes correct identifications while minimizing erroneous suspect identifications (Luus & Wells, 1991).

Although a great deal of research has examined the effect of suspect–filler similarity on identification performance (e.g., see Fitzgerald et al., 2013), these results are difficult to translate into practical advice for police procedure. For example, recent guidelines from the Department of Justice (DOJ) recommend selecting fillers that fit the general description of the offender, and are not too similar nor too dissimilar to the suspect (U.S. Department of Justice, 2017). However, it is unclear what “too similar” and “too dissimilar” actually mean. The DOJ goes further stating that the fillers “should be sufficiently similar so that a suspect’s photograph does not stand out, but not so similar that a person who knew the suspect would find it difficult to distinguish him or her.” Nevertheless, this recommendation still invites subjectivity, and therefore, its implementation will likely vary by individuals and precincts.

Perhaps because of the difficulty in defining similarity in a way that is more precise and objective, extant recommendations understandably focus on the *process* of filler selection rather than the specific desired *outcome* (Wells et al., 2000; see also Luus & Wells, 1991). This research argues that eyewitness identification is (generally) best served when the lineup is constructed with fillers matched to the witness’ description of the perpetrator rather than to the suspect themselves (Wells et al., 2000).

Although this recommendation provides concrete advice regarding filler selection, it is unclear whether a lineup with purely description-matched fillers will always lead to the optimal eyewitness performance (Tunnicliff & Clark, 2000). One problem with this recommendation is that it assumes a reasonably accurate and detailed description of the perpetrator (see Wells et al., 2020 on what to do when the description is inaccurate). However, there is evidence that witness descriptions are often missing important information, and that matching based on such descriptions can lead to elevated innocent suspect identification rates (Lindsay et al., 1994). In these cases, researchers recommend matching the fillers to the suspect on general characteristics, such as age, sex, and race (e.g., Wells et al., 2020).

In addition to the verbalizable features that witnesses sometimes omit, it is also important to consider the substantial category of features that do not easily lend themselves to being described. For example, there is a large body of evidence to suggest that face processing is holistic (e.g., Young et al., 2013), and therefore, not conducive to a feature-based process required to verbally describe a face (see also, Wells & Hryciw, 1984).

Further evidence arguing against the superiority of description-matched fillers is those studies which simply fail to demonstrate that such a strategy actually improves identification performance. For example, in two experiments, Tunnicliff and Clark (2000) compared the benefit of a lineup with description-matched fillers to a lineup with suspect-matched fillers. Despite using lineup constructors from different populations, performance was comparable between suspect-matched and description-matched lineups.^1^ Perhaps more convincingly, a recent meta-analysis examined how suspect–filler similarity affects identification performance (Fitzgerald et al., 2013). Collapsing across nine empirical studies, this analysis showed that lineups with fillers that were highly similar to the suspect yielded lower innocent suspect identification rates compared to a lineup with moderately similar fillers, but had no effect on correct identification rates. It should be noted that although suspect–filler similarity can be manipulated for lineups with description-matched or suspect-matched fillers, in Fitzgerald et al. (2013), lineups with suspect-matched fillers were consistently classified as having higher suspect–filler similarity than lineups with description-matched fillers.

Even if lineup administrators take the relatively straightforward advice of matching fillers to the witness’ description, ambiguity still persists after the features have been matched. That is, if a witness describes the perpetrator as a heavy set, tall male in his late forties, once those features have been matched, how closely (if at all) should the fillers resemble the suspect? Current best practices suggest that fillers should be substantially dissimilar from the suspect after matching to the description (Wells et al., 1993; see also Colloff et al., 2021). This highlights that the recommendation to match fillers to the description of the offender does not eliminate the burden of considering suspect–filler similarity in any systematic way. Put differently, this suggests that some blended approach of filler selection yields better discriminability than using either approach in isolation.

Indeed, there are potential benefits and costs of both types of filler selection approaches. The benefit of selecting fillers based on a witness’ description is that it provides an obvious stopping point for how similar the fillers should be (e.g., if the witness mentions three physical characteristics of the perpetrator, officers can match on those three dimensions and nothing more), and reduces the amount of subjectivity involved in the selection process. However, the potential costs of using description-matched fillers are that descriptions can be inaccurate, or sparse in detail, which may partly stem from the fact that faces are difficult to describe (e.g., see Frowd et al., 2005; Meissner et al., 2007). In addition, as mentioned above, there is evidence that the level of similarity that optimizes performance is less similar than lineups with purely description-matched fillers (Colloff et al., 2021; Wells et al., 1993). Of course, selecting fillers based on the appearance of the suspect is more subjective and provides no obvious stopping point for how similar the fillers should be, which may lead to biased lineups, if the fillers are not similar enough (see also Navon, 1992). In the current study, we use a blended approach by matching fillers on general characteristics and then further manipulating the similarity of the fillers to the suspect in a more objective fashion.

Looking broadly across the literature, generating clear recommendations about suspect–filler similarity is fraught because each study defines similarity idiosyncratically. Some researchers have attempted to approach operationalizing similarity by using morphing software which creates a new, artificial face from two or more seed faces. In this way, researchers can systematically vary how similar fillers are to the suspect by specifying exactly how much of the components of each seed are incorporated into the various composite faces. Using this approach, Fitzgerald et al. (2015) found that highly similar fillers yielded a lower correct identification rate compared to moderately similar fillers. This conclusion notably differs from these authors’ previous meta-analysis which indicated that the use of high-similarity fillers was not reliably associated with a reduction in correct identification rates. The authors speculated that the morphing software that was used allows for a much greater level of similarity than face photograph databases that are used by researchers. This suggests that the relationship between similarity and witness performance may be nonlinear. That is, problems arise when fillers are both too dissimilar *and* too similar.

One may intuit, then, there is an ideal zone of similarity in which the fillers are neither too dissimilar nor similar that maximizes witnesses’ ability to make correct identifications. The critical question, then, is: How do we define that zone? Historically, researchers have relied upon ordinal labels to characterize similarity, but that approach has led to the current ambiguous state reviewed thus far. A more objective approach to defining similarity would not only be beneficial for researchers comparing outcomes of various studies but also to policy makers and lineup administrators who could more easily apply prescriptive recommendations since those recommendations would not be reliant upon subjective decision making. Using face morphing software (e.g., Fitzgerald et al., 2015) might seem like a candidate solution due to its ability to objectively quantify how similar two or more faces are. However, as the authors noted, this procedure carries additional concerns of ecological validity. For example, this procedure requires one to use a relatively homogenous set of faces in order to yield fillers that do not appear to be morphs. Similarly, it is not obvious how identification performance is affected when all of the fillers are morphs of the suspect. This is because morphing would likely increase the familiarity of both the suspect and the fillers, and it is not clear that the increase would be comparable between the two types of photographs. It is also possible that the morphing procedure would yield more typical fillers, as a result of the averaging among faces.

The current study aims to measure similarity in a more precise fashion, using multidimensional scaling (MDS, e.g., Kruskal & Wish, 1978; Kruskal, 1964a, 1964b; Rabinowitz, 1975). MDS is an exploratory data analysis technique that provides a set of interitem distances in a *k*-dimensional space where *k* represents the number of dimensions that are specified by a given scaling solution. Importantly, the algorithm seeks to create a space in which perceived similarity is monotonically related to distance, among all the stimuli in the set. As a consequence, similarity can be measured such that stimuli are similar to the extent that they are closer in space (i.e., less distance). For example, imagine a hypothetical set of faces that vary on a number of dimensions, such as age, sex, and eye size. MDS attempts to determine which dimensions are most important in defining the similarity among the set of faces. In applying MDS to this hypothetical set of faces, one may find that two dimensions captures a sufficient amount of variation among the faces. Upon inspection of this face space, one may notice that faces varying on one dimension vary in skin tone, whereas faces that vary on the other dimension vary in age. As a result, the researcher may infer that the two dimensions of the face space are age and skin tone. MDS has been quite useful in measuring psychological similarity (e.g., Clark et al., 1986; Hout et al., 2016; Howard & Howard, 1977; Papesh & Goldinger, 2010; Shepard, 1980), particularly because it allows researchers to infer the specific dimensions by which the space is defined. As a result, this gives researchers some idea as to which dimensions are most important in defining similarity.

One limitation of MDS is that it often relies on data collection that is time-consuming and inefficient, as it requires participants to make pairwise comparisons among all possible pairs of stimuli (but see Goldstone, 1994; Hout et al., 2013 for alternative data collection methods). That is, for a stimulus set of *n* items, *n*(*n *− 1)/2 ratings are required. As such, a database of, for example, 100 faces necessitates 4950 ratings (Goldstone, 1994). Because the number of comparisons required can grow quite rapidly, it is impractical to solicit ratings for anything but small face databases.

Fortunately, human responses are not required to create an MDS face space; there are other approaches. Specifically, faces can alternatively be quantified by identifying specific landmarks (using computer software) of a given face (e.g., tip of the nose, the corners of the eyes and mouth, etc.). With these measurements, each face can be defined as a vector of numbers, which can be used to compute a measure of Euclidean distance for each pair of faces. These distances can then be used to construct an *n*-dimensional “face space” in a similar fashion as described earlier (albeit without the labor associated with gathering human-provided responses). By employing these objective measurements, we can circumvent the issues reviewed previously concerning the difficulties in operationalizing similarity. We should note that this approach is not completely objective in that it does not completely remove human judgment from the process. This is because researchers and investigators have some discretion over the amount and type of data that each face contributes. Nevertheless, we think that this approach provides a more objective way of defining similarity than much of the previous research.

While using this approach, we were able to use MDS to create a face space from a database of faces far larger than what would be possible using human ratings. In the current study, we gathered a set of 82,028 mugshots from which we extracted information about each face. From each face, we extracted information such as age, sex, and race, but the majority of the information extracted relates to facial landmarks (referred to as fiducials, see “Materials” section for more information). Using a database of this size is critical because it provides us with a large sample of photographs to choose from, which should allow us to find fillers that are *exceptionally* similar to the suspect. Some have suggested that because investigators often have access to a much larger database of photographs to select fillers from, relative to researchers, studies may not be observing this relationship at the higher end of the similarity scale (Bergold & Heaton, 2018; Fitzgerald et al., 2013, 2015). Therefore, a database of this size affords us more precise control over the degree of suspect–filler similarity. In addition, a database of this size will yield an MDS space that is more representative of how faces vary.

To the extent that our method of quantifying similarity accurately captures how faces are perceived (see Tredoux, 2002), this approach is useful in more precisely operationalizing similarity, which should prove useful in theory development and testing. For example, the recommendation to select fillers based on the description of the offender (Carlson et al., 2019; Colloff et al., 2021; Juslin et al., 1996; Lindsay & Wells, 1980; Luus & Wells, 1991; Navon, 1992; Technical Working Group, 2003; U.S. Department of Justice, 2017; Wells et al., 1993) was, in part, based on the notion that description-matched fillers will lead to lineups with “propitious heterogeneity” among lineup members (Luus & Wells, 1991), which should aid recognition (Gibson, 1969). Indeed, subsequent research found that dissimilar fillers that are otherwise matched to the description of the offender enhanced identification performance relative to suspect-matched lineups (Wells et al., 1993). More recently, the diagnostic-feature-detection (DFD) theory was introduced (Wixted & Mickes, 2014), stating that the discriminability of a procedure is determined by the extent to which it emphasizes which features are diagnostic. For example, when fillers are matched to the features in the description of the offender, these features are rendered nondiagnostic, which allows a witness to focus on the more diagnostic features. Subsequent modeling (Colloff et al., 2021) showed that the DFD theory predicts a benefit of dissimilar, description-matched fillers over more similar fillers, as Luus and Wells (1991) predicted. They also confirmed this prediction empirically by replicating Wells et al. (1993). Therefore, in the current study, we should find that lineups with the least similar fillers, within a given subset of mugshots generally matching on age, sex, and race, should yield the greatest discriminability. That is, in the current study, both the propitious heterogeneity and the DFD hypotheses predict that discriminability will be greatest for lineups with fillers that are on the lower end of the similarity scale, after matching on the general characteristics of the offender. These hypotheses were preregistered prior to any data collection.

In the current experiment, participants were shown a series of faces to study. After initial encoding, they completed a distractor task, followed by four lineups with the studied face (“guilty suspect”) or an unstudied face (“innocent suspect”) among 5 fillers. Each lineup was associated with only one of the studied faces. Importantly, the similarity between the suspect and fillers was varied. This design allows us to examine how suspect–filler similarity affects discriminability across a wide range of the similarity scale.

## Method

### Participants

A power analysis was conducted using the powe(R)OC app in R (Mah, 2022). This analysis indicated that 400 participants are required in order to detect an effect size of 0.15 with approximately 0.8 power. This effect size is equal to 1 − $\frac{{pAUC_{1} }}{{pAUC_{2} }}$ where $pAUC_{1}$ is greater than or equal to $pAUC_{2}$. This effect size value is based on the comparison between high-similarity fillers and low-similarity fillers from Colloff et al., (2021, Exp. 1). We recruited a total of 592 participants from Prolific to participate in this study. All of the participants were between 18 and 60 years old. A total of 18 participants gave nonsensical answers to our open question (i.e., “Please describe a past experience that you enjoyed.”), and therefore, their data were excluded. Data from an additional 168 participants were excluded because they either exited full screen or clicked outside of the browser on study and test trials. This resulted in a total of 406 participants. All of the data can be found here: https://osf.io/tb3hu/.

### Design

A 4 (Suspect–filler similarity: High, Medium–High, Medium–Low, Low) × 2 (Target presence: Present, Absent) within-subjects design was used in this study. There was only one lineup per studied face. There were a total of six blocks. For each block, there were four sequential study trials. In each trial, one face was studied. After a distractor task, there were four lineup trials. For each lineup trial, one of the four faces was tested on. Race and sex were random variables with the constraint that each combination was represented in each block once. Suspect–filler similarity and target presence were balanced across all six blocks (see Table 1 for the structure of an example list).

**Table 1:** Example list demonstrating the design of study

Condition	Block	Trial	Race	Sex	Age	Target
Low	1	1	Black	Woman	30	TP
Medium–low	1	2	White	Man	30	TA
Medium–high	1	3	White	Woman	20	TP
High	1	4	Black	Man	40	TP
High	2	1	White	Woman	30	TP
Low	2	2	White	Man	30	TP
Medium–low	2	3	Black	Man	20	TA
Low	2	4	Black	Woman	30	TA
Low	3	1	White	Man	20	TP
Low	3	2	Black	Woman	30	TA
Medium–high	3	3	Black	Man	20	TA
High	3	4	White	Woman	30	TA
Medium–high	4	1	Black	Man	20	TA
Low	4	2	White	Woman	20	TA
High	4	3	Black	Woman	40	TP
High	4	4	White	Man	20	TA
Medium–high	5	1	White	Man	20	TA
Medium–low	5	2	Black	Man	30	TP
Medium–low	5	3	White	Woman	40	TP
Medium–high	5	4	Black	Woman	20	TP
Medium–low	6	1	White	Man	40	TA
High	6	2	Black	Woman	30	TA
Medium–high	6	3	White	Woman	20	TP
Medium–low	6	4	Black	Man	20	TP

*TP* target-present, *TA* target-absent

### Materials

**Fig. 1:**
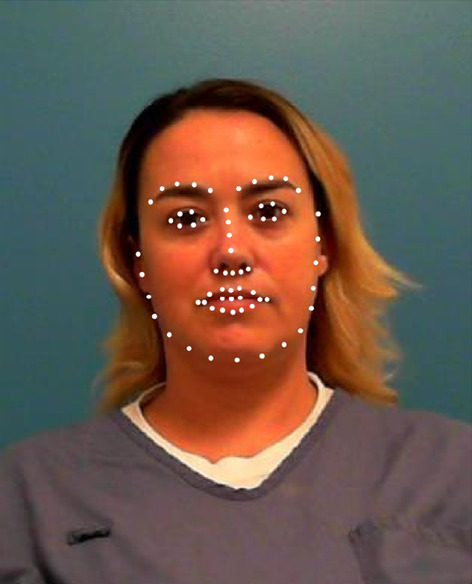
One face with their facial landmarks overlaid on their face in white dots. The *x*- and *y*-coordinates of the face as used in the MDS solution, along with: their actual age, their predicted age, numbers corresponding to the prediction of how likely the face belongs to each of the following races (i.e., Asian, Indian, Black, White, Middle Eastern, and Hispanic). *Note*: the actual landmarks have been increased from their pixel-length diameter for viewing purposes. There are 68 points, but some of the points overlap, giving the appearance of only 64

We downloaded 82,028 mugshots from the publicly available offender database maintained by the Florida Department of Corrections (FL DoC; http://www.dc.state.fl.us/). We also downloaded information about the age of each offender at the time in which the mugshot was created, as well as the sex and race of each offender. From these, we used the mugshots of offenders at least 20 years of age, but no older than 49 years of age. Finally, we only used black and white males and females (the lower quantity of faces in other racial categories and the lack of information about nonbinary gender precluded a broader examination). In order to ensure that the resolution and size of all of the pictures were similar, we only used pictures that were at least 380 pixels in length and had an aspect ratio of at least 1.2. In doing so, there were 1116 black females, 27,535 black males, 2369 white females, and 22,425 white males. For each of these images, we used OpenCV, a Python library (Bradski, 2000; see https://github.com/opencv) to extract facial landmarks, as well as make predictions about the person’s age, sex, race, and emotion. We included only facial landmarks, the person’s predicted age, and race because the program did a poor job at predicting sex and this was not expected to vary within a subset of mugshots. We also did not include information on emotion because this characteristic is not inherent to the person’s appearance. For each image, we used 136 facial landmarks (i.e., 68 pairs), their actual age, their predicted age, and five values, each pertaining to the probability that a given face is Asian, Indian, Black, Middle Eastern, White, and Latin (see Fig. 1 for an example). These sets were further divided into three bins: individuals aged 20–29 years old, 30–39 years old, and 40–49 years old. This was done for all of the categories. Table 2 shows the frequencies of each set. For each Race × Sex × Age subgroup, we created a 10-dimensional space. There is no strong consensus on how many dimensions should ideally be implemented, as researchers have argued that this number is anywhere from three to 70 (see Lewis, 2004). Some studies have argued for a lower range (three to six dimensions; Busey, 1998; Lee, et al., 2000; Rhodes, 1988; Steyvers & Busey, 2000), whereas others arguing for a much larger range (10+; see Lewis, 2004 for a discussion). Our decision to use ten dimensions was based on a desire to specify a dimensionality that led to a maximal reduction in error variance, but also yielded solutions that fit each subgroup similarly.

**Table 2:** Frequencies of mugshots used, as a function of race, sex, and age group

Race	Sex	Age group			
		20–29	30–39	40–49	Total
Black	Males	8673	11,248	7614	27,535
	Females	396	441	279	1116
White	Males	4999	9652	7774	22,425
	Females	514	1101	754	2369
	Total	14,582	22,442	16,421	53,445

**Fig. 2:**
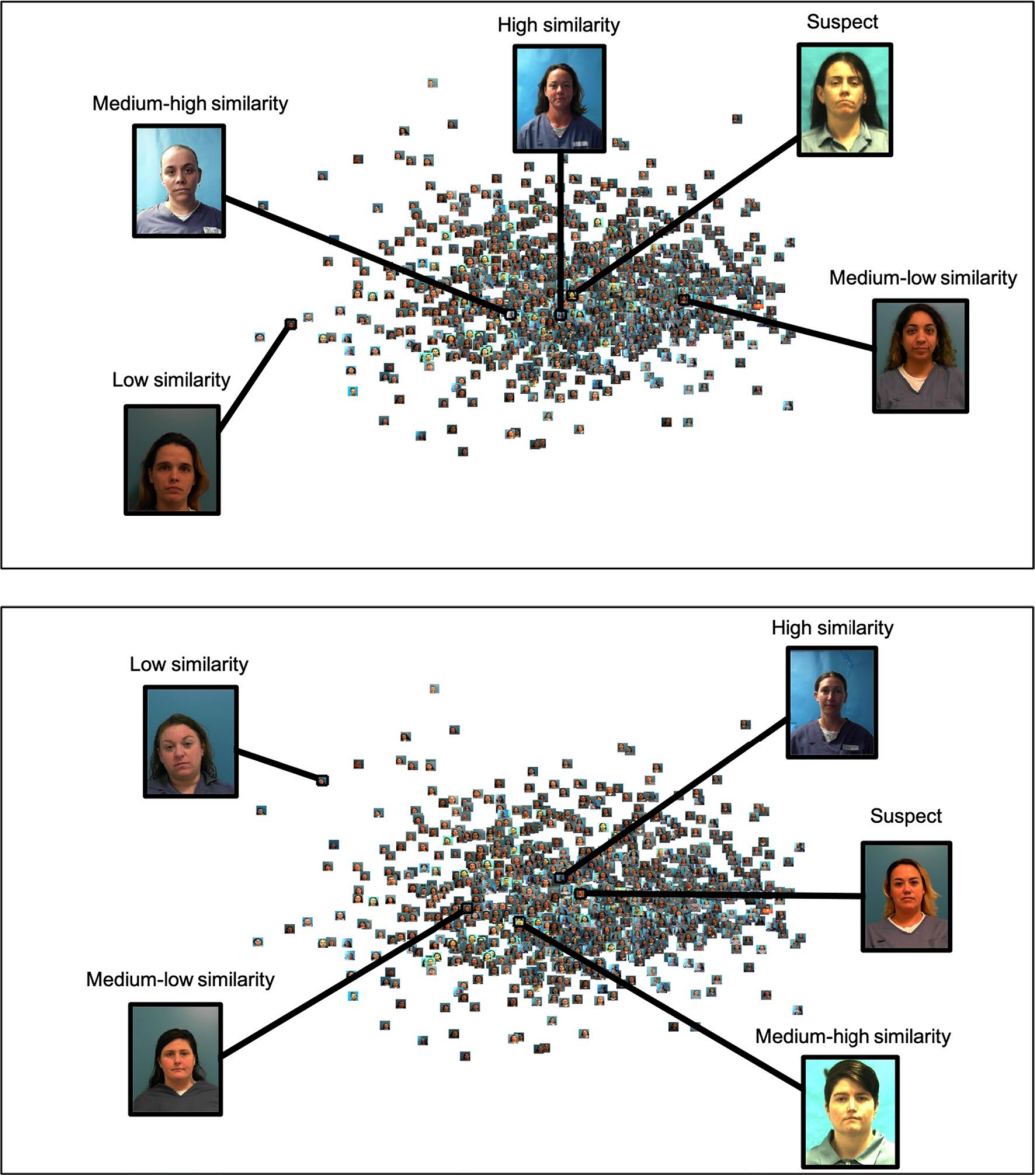
A two-dimensional face space of white females in their 30 s. Each panel shows an example of a suspect, with a potential filler for each level of similarity, based on distance from the suspect. The top panel shows an example using a randomly determined guilty suspect. The bottom panel shows an example using a randomly determined innocent suspect. *Note*: Two dimensions were specified for these spaces for illustrative purposes only

For each participant, we created 24 lineups. For each target-absent (TA) trial, we randomly chose two images. One of these images would be studied, and one would serve as the innocent suspect. For each target-present (TP) trial, we randomly chose only one image which served as a studied item and included in the subsequent lineup as a guilty suspect. A photograph of a guilty suspect was identical to the photograph of a studied item. For each set of four lineups, we chose images from a randomly selected age group, for each sex by race combination, without replacement. For each trial, the fillers for each lineup were selected based on their similarity to the suspect (i.e., their distance from the suspect in the MDS space). In the high-similarity trials, the five photographs closest to the suspect were selected to be fillers. In the medium–high-similarity trials, photographs that were closer to the suspect than around 66% of the photographs were selected to be the fillers. In the medium–low-similarity trials, photographs that were closer to the suspect than around 33% of the photographs were selected to be the fillers. Finally, in the low-similarity trial, the five photographs that were the farthest from the suspect were chosen to be the fillers (see Fig. 2). For subsequent trials within a given Race × Sex × Age subgroup, all of the used photographs were removed from the pool of available mugshots prior to selecting fillers. Across all 24 lineups, there was an equal number of lineups from each race by sex combination. Each study and lineup trial were randomly assigned to one of six blocks with the constraint that each sex by race combination was represented once.^2^

## Procedure

Participants studied six blocks. For each block, four photographs were presented sequentially for 1 s each.^3^ Each block contained one Black woman, one Black man, one White woman, and one White man. After studying all four photographs, participants were given a distractor task (i.e., Tetris) for 60 s. Following this task, participants were given four lineups, one after the other. Photographs of guilty suspects were identical to photographs of studied items. For each lineup, they were asked “Is the white (or black) woman (or man) from the last four photographs present in the lineup?.” They were further told that the person from study may or may not be present in the lineup, and to select them if they are present, and reject the lineup if they are not present in the lineup. After each response, they were then asked to give a confidence rating in their decision, on a scale from 0 to 100. For each block, the order of test position matched the order of the study position. The order of study position was randomized for each block.

## Results

All analyses were performed in R (R Core Team, 2021).

### Discriminability

Our research question was how suspect–filler similarity affects discriminability. To answer this, we conducted ROC analyses. As an additional planned analysis, as specified by our preregistration, we also fit signal detection theory models to the data; however, we chose to move them to Supplementary Materials (see Additional file 1: Tables S1–S2), because we realized that the Independent Observation (IO) model is not appropriate (see Shen et al., 2023).^4^ In addition, the resulting model fits were poor. As such, we do not to discuss these results. We first compared whether the two medium-similarity conditions differed from each other. If they did not differ, we collapsed the data from these two conditions, in order to reduce the number of pairwise comparisons (with Bonferroni corrections) from six to four. Regardless, for all figures, we show the results of all four conditions. Because we are predicting that fillers that are less similar to the suspect will result in improved discriminability, each comparison was a one-tailed test. Each participant contributed three observations to each target presence × similarity combination. Therefore, all analyses were done on the disaggregated data. That is, the pAUCs could not be computed for each participant, and the data were treated as though each observation contributes to a condition, rather than to a participant. For the ROC analysis, the false alarms were based on instances in which the designated innocent suspect was chosen.

**Fig. 3:**
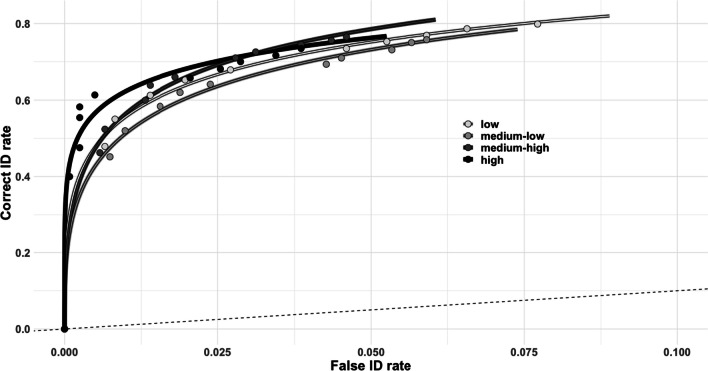
Receiver operating characteristic (ROC) plot for each suspect–filler similarity condition. Dashed line indicates chance performance

We conducted ROC analysis, using the pROC package (Robin et al., 2011), and compared *partial area under the curve* (pAUC) between suspect–filler similarity conditions. Each curve plotted 11 hit rate (HR) and false alarm rate (FAR) pairs over decreasing levels of confidence. The first HR-FAR pair corresponds to cases in which participants responded with 100% confidence. The second HR-FAR pair corresponds to cases in which participants responded with 90% or more confidence. This continues up to the rightmost points of the curves which corresponds to the cumulative HR and FAR, and includes cases in which participants choose a suspect, regardless of the confidence rating that was provided. For statistically comparing the pAUCs of each condition, we truncated the ROCs to the condition with the lowest cumulative false alarm rate. We should note that when comparing conditions, we used a paired statistical test. However, when the two medium-similarity conditions were collapsed and compared to other similarity conditions, a paired test was not possible. This is because the pROC package does not appear to allow for an unequal number of observations for paired statistical tests. For completeness, we report the ROC curves in Fig. 3 for all filler similarity conditions, regardless of whether the medium-similarity conditions differed from each other.

The medium–low-similarity condition (pAUC = 0.021) did not differ from the medium–high-similarity condition (pAUC = 0.023), *D* = − 1.44, *p* = 0.92. Therefore, we collapsed the data from these two conditions. The low-similarity condition (pAUC = 0.022) did not differ from the medium-similarity conditions (pAUC = 0.022), *D* = 0.25, *p* = 0.40, nor from the high-similarity condition (pAUC = 0.025), *D* = − 1.69, *p* = 0.95. Finally, the medium-similarity conditions did not differ from the high-similarity condition, *D* = − 2.40, *p* = 0.99. Contrary to our predictions, it appears that suspect–filler similarity did not affect discriminability. However, the high-similarity condition does show the largest pAUC (see Fig. 3).

### Exploratory analyses

Based on our planned analysis, we did not find any effect of suspect–filler similarity on discriminability. However, we should note that one likely reason for this is that we used a one-tailed statistical test that predicted that the condition with the lower similarity would outperform the higher-similarity condition. This decision was based on the assumption that all of our lineups would be fair, as the fillers all matched on general characteristics, such as age, sex, and race. We should mention that even though the IO model is not appropriate, it often fits the data about as well the Ensemble model does (Shen et al., 2023; Wixted et al., 2018). Therefore, given the unusual values of *d*_*a*_, and the poor fits (see Additional file 1: Tables S1–S2), we suspected that the assumption of lineup fairness may not have been met, and that some of our lineups may have varied in terms of how fair they were. We computed the conditional probability of choosing the innocent suspect, given that any lineup member was chosen out of a TA lineup. All of these values were close to, and did not statistically exceed 0.167, with the exception of the low-similarity condition, with a conditional probability of 0.30, *z* = 6.29, *p* < 0.001. As an exploratory analysis, we examined this further by fitting signal detection theory models in which the mean of the innocent suspect distribution was allowed to vary (Cohen et al., 2021).^5^ However, for the same reasons as above, this model is also not appropriate, as it also predicts no effect of suspect–filler similarity on *theoretical* discriminability. Therefore, we also include these in Supplementary Materials (see Additional file 1: Table S3). We also fit the Ensemble model to our data using *pyWitness* (Mickes et al., 2022).^6^ This software does not yield parameter estimates for the base-rate parameter, nor rejection criteria. These models also assume that the lineups were fair. In addition, the bootstrapping procedure takes a prohibitively long time. As a result, we were not able to statistically compare conditions. We do not discuss these results, but include the model fits in Supplementary Materials (see Additional file 1: Table S4).

**Fig. 4:**
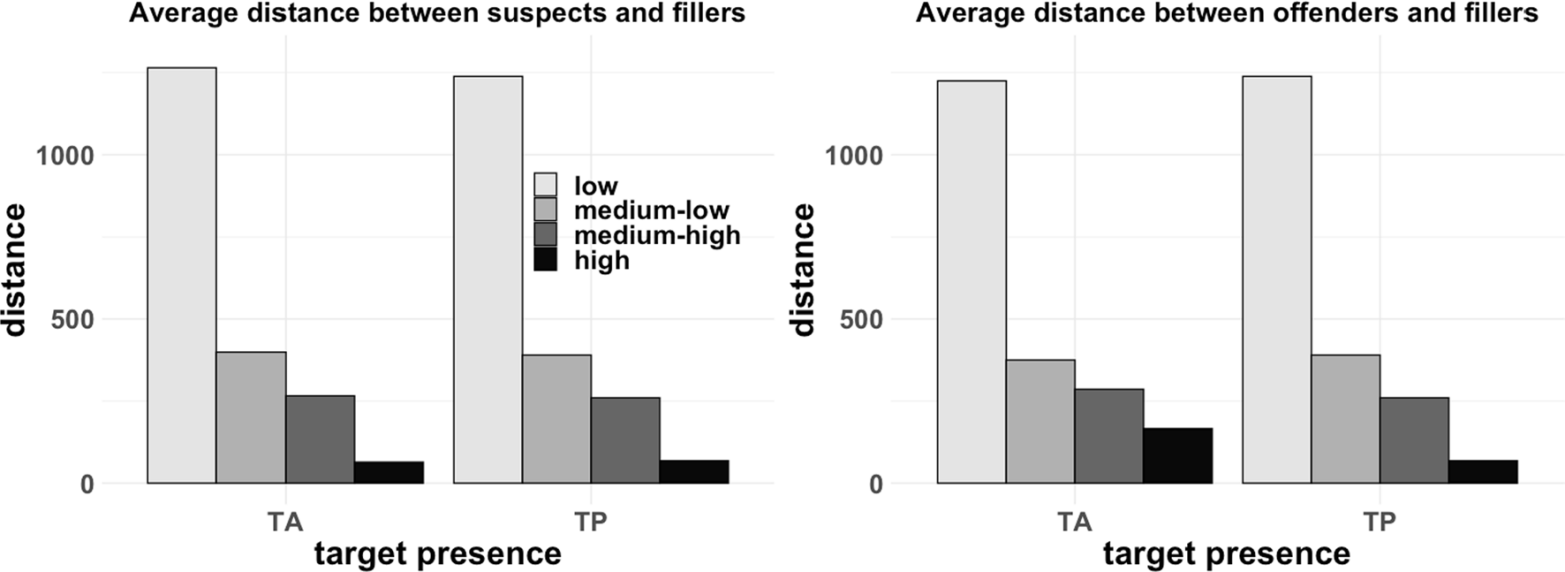
Left panel: Average distance between suspect and fillers as a function of similarity and target presence. Right panel: Average distance between offenders and fillers as a function of similarity and target presence. The distance between offenders and fillers in TP lineups is identical to the distance between suspects and fillers in TP lineups

**Fig. 5:**
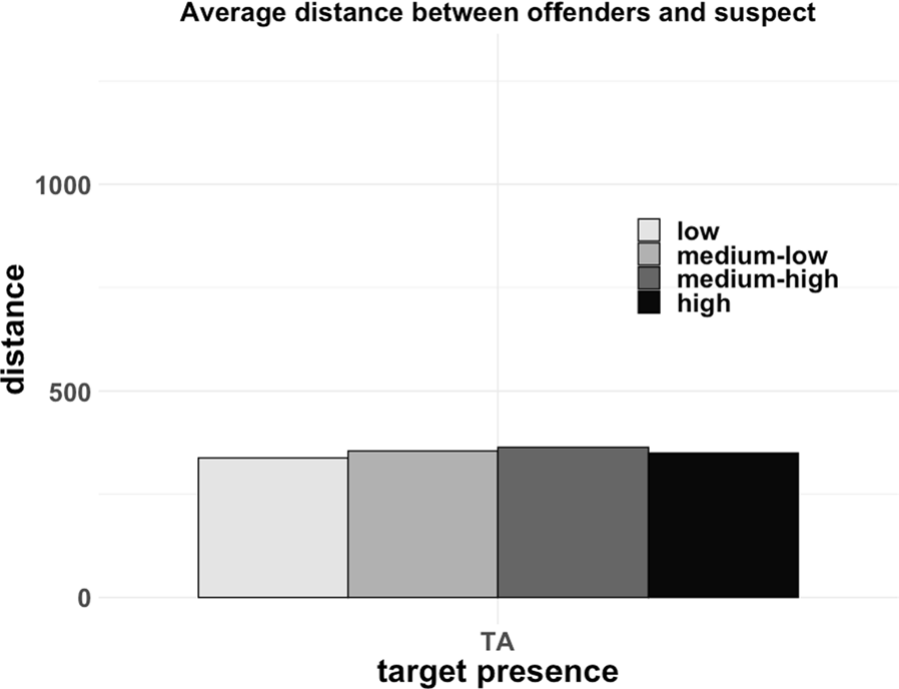
Average distance between offenders and innocent suspects as a function of filler similarity

To further examine whether the conditions varied in lineup fairness, for each trial, we mapped the innocent suspect, the guilty suspect, and the fillers onto its respective MDS space. This analysis was exploratory. Based on these spaces, we computed the average distance between offenders and fillers, as well as between innocent suspects and fillers, as a function of target presence and suspect–filler similarity conditions. We also computed the average distance between offenders and innocent suspects as a function of suspect–filler similarity. As shown in the left panel of Fig. 4, the distance between suspects and a set of fillers decreased as a function of suspect–filler similarity, which was similar for target-present and target-absent lineups. In the right panel of Fig. 4, the distance between an offender and a set of fillers decreased as the suspect–filler similarity increased. That is, as the fillers became more similar to an innocent suspect, they also became more similar to the guilty suspect. This occurred despite that fact that the similarity between a guilty and innocent suspect was comparable across conditions (see Fig. 5). Interestingly, these data suggest that because the innocent and guilty suspect were more likely to be concentrated toward the center of the face space, the similarity that they had with a filler from either lineup was likely to be comparable. That is, when both the innocent and guilty suspect were relatively typical in their appearance, the fillers that were chosen based on their similarity to the innocent suspect were often of a comparable similarity to the guilty suspect. As shown in Fig. 5, the distance between innocent and guilty suspects was within the range of distances between suspect and fillers in the medium-similarity conditions. In other words, it appears that the medium-similarity fillers were as similar to the innocent suspect as they were to the offender. Therefore, despite matching fillers to suspects on general characteristics such as race, sex, and age, it is possible that the conditions varied in lineup fairness. This would stem from the fact that our innocent and guilty suspects were randomly sampled from a distribution in which typical faces are more densely populated. As a consequence, these faces are more likely to be selected as suspects than more distinctive faces. This means that innocent and guilty suspects will often be similar to each other.

### Confidence–accuracy characteristic curves

**Fig. 6:**
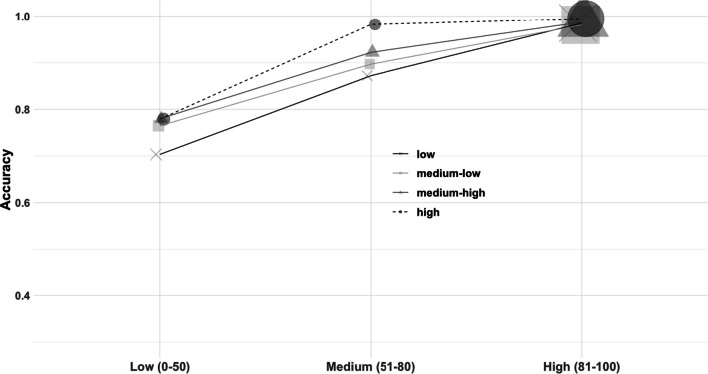
Confidence–accuracy characteristic (CAC) curve as a function of filler similarity condition. Accuracy = [correct IDs/(correct IDs + incorrect suspect IDs in the TA lineup)]. Marker size is based on the number of participants that contributed to that cell

Although it was not our focus, we constructed confidence–accuracy characteristic (CAC) curves for each condition to examine whether suspect–filler similarity affects the confidence–accuracy relationship. This analysis was exploratory. For each similarity condition, the correct suspect identification rate (i.e., correct suspect identifications / (correct suspect identifications + incorrect suspect identifications)) were computed for a confidence of 0–50, 51–80, and 81–100.^7^ As shown in Fig. 6, accuracy appeared to increase as a function of confidence, for all the filler similarity conditions. To statistically test whether confidence was related to accuracy within each condition, we used a bootstrapping procedure to compute the sampling distribution of a given difference in accuracy between two given bins. The 95% confidence intervals of the difference in accuracy between two given bins (e.g., low confidence vs. medium confidence) for each condition are presented in Table 3. With only one exception of the twelve comparisons, accuracy was greater in the higher confidence bin. For exploratory purposes, we also repeated all the planned analyses above for each Race $\times \hspace{0.17em}$Sex combination. This was done because the number of mugshots in each subgroup differs substantially. For example, there are more than twice as many white females as there are black females, and there are more than fourteen times more males than females. Therefore, it is quite likely that a greater level of similarity can be achieved when lineups are constructed when sampling from a larger number of mugshots (Bergold & Heaton, 2018). However, for the sake of space, we report these results in Supplemental Materials (see Additional file 1: Table S5–S21). In general, the findings of each Race × Sex subgroup were consistent with the aggregate data set.^8^

**Table 3:** Confidence intervals of the difference in accuracy between each confidence bin within each filler similarity condition

*Low*		
Bin 1	Bin 2	95% CI
Low	Medium	[− 0.23, − 0.09]
Low	High	[− 0.33, − 0.24]
Medium	High	[− 0.16, − 0.07]
*Medium–low*		
Bin 1	Bin 2	95% CI
Low	Medium	[− 0.21, − 0.06]
Low	High	[− 0.26, − 0.17]
Medium	High	[− 0.13, − 0.04]
*Medium–high*		
Bin 1	Bin 2	95% CI
Low	Medium	[− 0.21, − 0.07]
Low	High	[− 0.26, − 0.16]
Medium	High	[− 0.10, − 0.03]
*High*		
Bin 1	Bin 2	95% CI
Low	Medium	[− 0.24, − 0.16]
Low	High	[− 0.25, − 0.18]
Medium	High	[− 0.03, 0.00]

## Discussion

The current study manipulated suspect–filler similarity by constructing a multidimensional scaling model based on facial metadata and selecting fillers on the basis of distance from the suspect. Based on the notion of “propitious heterogeneity” (Wells et al., 1993), and the diagnostic-feature-detection (DFD) theory (Colloff et al., 2021), we predicted that discriminability would be the greatest for lineups with low suspect–filler similarity. This prediction was not supported by the data. Based on the ROC analysis, none of the conditions differed from each other, though this is likely because we used directional statistical tests, which predicted the opposite of what we found. That is, we predicted a benefit of lower-similarity fillers, yet the high-similarity condition was numerically larger than the other conditions.

However, as mentioned above, both the DFD theory and the notion of propitious heterogeneity assert that low similarity is beneficial, but only for fair lineups. According to this definition, a lineup is fair if the fillers are matched to the description of the offender. In the current study, we did not choose fillers based on a participant’s description, and instead, we matched fillers based on general demographic characteristics. We assumed that our method was a reasonable approximation to choosing based on the description. However, given that the conditional probability of choosing the innocent suspect exceeded chance in the low-similarity condition, it is possible that this assumption was not met.

We expected that the average similarity between innocent and guilty suspects would likely be most comparable to the average suspect–filler similarity of the medium-similarity conditions. This is because the innocent and guilty suspects were randomly selected from face spaces in which many of the faces were densely populated around the center. Indeed, this was corroborated by examining the average distance between suspects and fillers, as well as the average distance between innocent and guilty suspects.

The fact that this occurred suggests that fillers will be comparably similar to both the innocent and guilty suspects in cases which the two are relatively typical in their appearance. In other words, when the two suspects are typical in appearance, increasing the similarity of fillers to an innocent suspect may also increase the similarity of those fillers to a guilty suspect. In cases in which the guilty suspect is typical looking, the innocent suspect would also likely be more typical looking. Therefore, this natural confound is likely a characteristic of actual investigations.

This underscores the importance of considering the distinctiveness of a suspect when selecting fillers. For instance, when the suspect is distinctive, it will be more difficult to achieve a moderate or high level of suspect–filler similarity than when the suspect is more typical-looking. Conversely, it will be easier to find dissimilar-looking fillers when the suspect is distinctive, compared to when the suspect is more typical in appearance. Of course, this likely does not apply to situations in which a (guilty) suspect is distinctive because of an articulable feature, such as a facial scar. However, the characteristics of what makes a face distinctive may not always be articulated in an eyewitness’ description.

Given that we did not find any effect of suspect–filler similarity on discriminability, one might argue that our approach in manipulating similarity was not valid. However, there are few reasons why we believe that this is unlikely. First, the HRs decreased as a function of similarity (see Table 4). As discussed above, the potential cost of similar fillers is that, at some point, they will likely also reduce guilty suspect identifications. The fact that suspect–filler similarity reduced HRs suggests that our approach was valid. Second, because we randomly sampled innocent and guilty suspects, we expected that the similarity between these two suspects would be moderate, on average. Therefore, we would expect that the two medium-similarity conditions would yield lineups that were fair. This expectation was corroborated by the observation that in these conditions, the distance between suspects and fillers was comparable to the distance between the innocent and guilty suspects. Overall, our data suggest that our approach did effectively manipulate suspect–filler similarity.

**Table 4:** Frequencies of responses by lineup type and filler similarity condition

	SID	FID	No ID
*High*			
TP	895	94	229
TA	48	307	863
Total	943	401	1092
*Medium–high*			
TP	935	95	188
TA	59	304	855
Total	994	399	1043
*Medium–low*			
TP	924	93	201
TA	73	322	823
Total	997	415	1024
*Low*			
TP	974	59	185
TA	95	222	901
Total	1069	281	1086

*TP* target-present, *TA* target-absent, *SID* suspect identification, *FID* foil identification from the target-absent lineup, *No ID* no identification

It should be noted that the FARs in our study decreased as a function of suspect–filler similarity. Of course, because the potential benefit of similar fillers is that they should reduce innocent suspect identifications, this is not entirely surprising. However, it should be noted that the DFD theory predicts that FARs will decrease as function of suspect–filler similarity, but only when the similarity between innocent and guilty suspects is high (Colloff et al., 2021). When the innocent–guilty suspect similarity is moderate, then FARs should not be affected by suspect–filler similarity. When the innocent–guilty suspect similarity is low, FARs should increase as a function of suspect–filler similarity. Although we expected that on average, the innocent–guilty suspect similarity would be moderate, it is possible that they were more on the higher end of the similarity scale.

### Limitations and future directions

To manipulate suspect–filler similarity in the current study, we used an MDS model using metadata from each face. One general limitation of the current study is that in constructing MDS solutions, we took a relatively a theoretical approach. For example, we did not attempt to determine the psychologically correct number of dimensions that should be specified. As such, it is possible that we used an inappropriate number of dimensions. In addition, the MDS solution treated all of the information equally. This was done because we had no a priori reason to prioritize certain information over others. However, given that participants likely rely more heavily on certain characteristics when encoding faces, it is quite likely that some facial information is more important in determining similarity. It is possible that the MDS solutions prioritized dimensions that were not as important to participants. Similarly, we also did not transform the metadata in any way, apart from standardizing each measure. It is possible that the metadata could have been used to compute more informative measures. For example, the metadata included facial landmarks. However, it may have been more useful to use these facial landmarks to compute measures such as distance between the eyes and mouth width. The current approach to filler selection may be improved by using the metadata to derive such measures.

Similarly, the images were not standardized with respect to distance from the camera, for example, which may have contributed some amount of noise. Of course, from an applied perspective, mugshots are likely to vary on this dimension. However, this distance variable likely affected the facial landmarks that were extracted. That is, the landmarks from a face that is close to the camera will be more expanded than the landmarks from a face that is farther away from the camera. By preprocessing the images prior to extracting facial landmarks, it may be possible to get face spaces that are less noisy. Therefore, the filler selection process may be improved by preprocessing the images prior to extracting facial landmarks.

In addition, some of the people in the mugshots had face or neck tattoos, which could have influenced identification performance. Our goal was to see whether we could automate the filler selection process using objective measures of each mugshot. However, we were not able to automate the process of identifying mugshots with tattoos. Although the data suggest that we were able to manipulate suspect–filler similarity despite this fact, this method would likely be improved by filtering out mugshots with obvious tattoos.

A more obvious limitation of the current study is that we used the same mugshot during study and test. In applied scenarios, the situation is much more dynamic. The offender of a crime will likely be perceived from various angles, and the appearance of the offender during the crime will likely differ somewhat from the appearance of the offender in the lineup. Although it is not entirely clear that this would have changed the present results, it is still an important component that is lacking in this study. One possibility is that participants’ memory for the offender was sufficiently strong enough to identify the same photograph of a given person, and thus, less affected by our manipulation of suspect–filler similarity (Carlson et al., 2023). As such, the current approach may be improved by using different images of the offenders between encoding and test, and should be replicated with different images.

The current study attempted to manipulate suspect–filler similarity in a lineup via a novel method of using objective facial measures to generate face spaces. We believe that this first attempt was reasonably successful, as suggested by the fact that the HRs and FARs decreased with suspect–filler similarity. However, this does not mean that this approach cannot be improved upon by addressing the limitations of this approach, as currently implemented. In addition, it should be noted that the effectiveness of using MDS to manipulate suspect–filler similarity will likely depend on what kind of data the MDS solutions are based on. Of course, the ideal study would use psychological similarity ratings collected from humans as a basis for an MDS solution, but this may not be feasible given the practical and financial constraints of this data collection method. However, it may be possible to use these objective measures to compute features that are particularly important in face processing, which could then be used to generate an MDS solution.

When the witness of a crime is brought into a police precinct and shown a lineup, investigators must choose fillers to include in that lineup. Selecting fillers that are too dissimilar to the suspect may lead to a lineup in which an innocent suspect stands out from the fillers, which would likely put the innocent suspect at a greater risk of being falsely identified. Conversely, fillers that are too similar to the suspect may make it prohibitively difficult, rendering a correct identification less likely. Therefore, it is important to consider the level of suspect–filler similarity that would minimize the rate of innocent suspect identifications, while also maximizing the rate of guilty suspect identifications. Contrary to expectation, the current study found no evidence that suspect–filler similarity affected the extent to which innocent and guilty suspects were discriminated.

### Supplementary Information

**Additional file 1**. Supplementary tables.

## Data Availability

All of the data are available on OSF. Stimuli will not be provided due the potential ethical issues of increasing the accessibility of mugshots of prisoners.

## Footnotes

1 It is worth mentioning that in both experiments, discriminability, as measured by d' showed an advantage for suspect-matched lineups, though the methods were not statistically compared using this measure.

2 Across all blocks, suspect–filler similarity was crossed with target presence. However, block was not crossed with either of these variables, and neither of these variables were crossed with race and sex. See Table 1 for an example design.

3 Pilot work was done: first using 5 s and then using 3 s. In both cases, performance was relatively high. In response, we used a duration of 1 s per study face.

4 Although these model fits were part of our analysis plan in our original registered report (prior to data collection), we later realized that the independent observations (IO) model that the sdtlu package fits does not predict any effect of suspect–filler similarity on *theoretical* discriminability. Therefore, any differences may simply be due to excessive model flexibility (see Shen et al., 2023).

5 We thank Andrew Cohen for providing code with this modification.

6 We thank Laura Mickes and Stewart Boogert for assisting us with pyWitness.

7 The cutoffs for the confidence bins that were reported in original Registered Report were errors.

8 For the CAC analyses, we did not statistically compare accuracy between bins because there were often bins that had no innocent suspect identifications.
