# Supplementary material for: Using objective measures to examine the effect of suspect-filler similarity on eyewitness identification performance - Final Registered Report
Source: Cogn Res Princ Implic. 2023 Nov 6;8:68. doi: 10.1186/s41235-023-00522-w (PMC10628061; doi:10.1186/s41235-023-00522-w)
Supplement: Supplementary file 1 — Additional file 1. Supplementary tables. [file 41235_2023_522_MOESM1_ESM.docx]

Table S1. Unequal-variance signal (UVSD) detection model parameters and fits for each filler similarity condition.

|  | low | medium-low | medium-high | high |
| --- | --- | --- | --- | --- |
| *d_a_* | [9.61, 10.64, 11.76] | [7.77, 8.55, 9.46] | [8.90, 9.81, 10.84] | [7.64, 8.44, 9.30] |
| $\mu_{T}$ | 1.96 | 1.83 | 1.86 | 1.87 |
| $\sigma_{T}$ | 0.19 | 0.22 | 0.19 | 0.22 |
| $c_{1}$ | 1.79 | 1.66 | 1.7 | 1.71 |
| $c_{2}$ | 2.05 | 1.94 | 1.96 | 1.97 |
| $c_{3}$ | 2.13 | 2.03 | 2.05 | 2.05 |
| $c_{4}$ | 2.26 | 2.18 | 2.19 | 2.21 |
| $c_{5}$ | 2.38 | 2.34 | 2.33 | 2.35 |
|  |  |  |  |  |
| $p$ | 0.5 | 0.5 | 0.5 | 0.5 |
|  |  |  |  |  |
| $G^{2}$ | 455.9 | 389.15 | 335.57 | 402.71 |

Table S2. Equal-variance signal (EVSD) detection model parameters and fits for each filler similarity condition.

|  | low | medium-low | medium-high | high |
| --- | --- | --- | --- | --- |
| $\mu_{T}$ | [1.88, 1.95, 2.02] | [1.71, 1.77, 1.83] | [1.75, 1.81, 1.87] | [1.73, 1.79, 1.85] |
| $\sigma_{T}$ | 1.00 | 1.00 | 1.00 | 1.00 |
| $c_{1}$ | 1.6 | 1.49 | 1.52 | 1.55 |
| $c_{2}$ | 2.27 | 2.11 | 2.14 | 2.11 |
| $c_{3}$ | 2.46 | 2.28 | 2.34 | 2.28 |
| $c_{4}$ | 2.76 | 2.58 | 2.63 | 2.6 |
| $c_{5}$ | 3.04 | 2.89 | 2.9 | 2.91 |
|  |  |  |  |  |
| $p$ | 0.50 | 0.50 | 0.50 | 0.50 |
|  |  |  |  |  |
| $G^{2}$ | 1608.48 | 1454 | 1523.15 | 1352.53 |

Table S3. Unequal-variance signal (UVSD) detection model parameters and fits for each filler similarity condition, allowing for unfair lineups.

|  | low | medium-low | medium-high | high |
| --- | --- | --- | --- | --- |
| *d_a_* | [1.96, 2.11, 2.27] | [2.00, 2.15, 2.30] | [2.09, 2.26, 2.41] | [1.80, 2.00, 2.23] |
| $\mu_{T}$ | 2.89 | 2.55 | 2.62 | 2.47 |
| ${}_{T}$ | 1.30 | 1.27 | 1.27 | 1.25 |
| $\mu_{I}$ | 0.53 | 0.00 | 0.00 | 0.44 |
| ${}_{I}$ | 0.90 | 1.07 | 1.00 | 0.70 |
| $c_{Rej4}$ | 0.98 | 0.81 | 0.75 | 0.87 |
| $c_{Rej3}$ | 1.22 | 1.01 | 1.01 | 1.06 |
| $c_{Rej2}$ | 1.46 | 1.28 | 1.29 | 1.31 |
| $c_{Rej1}$ | 1.58 | 1.41 | 1.42 | 1.44 |
| $c_{ID1}$ | 1.75 | 1.56 | 1.58 | 1.60 |
| $c_{ID2}$ | 1.88 | 1.69 | 1.72 | 1.73 |
| $c_{ID3}$ | 2.04 | 1.85 | 1.86 | 1.88 |
| $c_{ID4}$ | 2.36 | 2.17 | 2.17 | 2.22 |
| $c_{ID5}$ | 2.72 | 2.47 | 2.53 | 2.53 |
|  |  |  |  |  |
| $p$ | 0.50 | 0.50 | 0.50 | 0.50 |
|  |  |  |  |  |
| $G^{2}$ | 15.41 | 30.99 | 19.84 | 41.11 |

Table S4. Ensemble model parameters and fits for each filler similarity condition, using pyWitness.

|  | low | medium-low | medium-high | high |
| --- | --- | --- | --- | --- |
| *d_a_* | 2.07 | 1.96 | 2.11 | 1.77 |
| $\mu_{T}$ | 1.06 | 1.18 | 1.01 | 0.74 |
| $\sigma_{T}$ | 0.68 | 0.80 | 0.60 | 0.55 |
| $\mu_{L}$ | -0.00 | -0.01 | -0.01 | -0.00 |
| $\sigma_{L}$ | 0.25 | 0.31 | 0.32 | 0.21 |
| $c_{1}$ | 0.40 | 0.47 | 0.47 | 0.32 |
| $c_{2}$ | 2.15 | 2.94 | 2.94 | 2.06 |
| $c_{3}$ | 2.36 | 2.94 | 3.07 | 2.06 |
| $c_{4}$ | 2.36 | 2.98 | 3.07 | 2.06 |
| $c_{5}$ | 2.36 | 2.98 | 3.09 | 2.07 |
|  |  |  |  |  |
| $G^{2}$ | 34.73 | 19.62 | 32.62 | 9.78 |

Table S5. Unequal-variance signal (UVSD) detection model parameters and fits for each filler similarity condition for black males only.

|  | low | medium-low | medium-high | high |
| --- | --- | --- | --- | --- |
| *d_a_* | [7.56, 9.19, 11.15] | [7.21, 8.93, 11.28] | [6.85, 8.43, 10.23] | [5.99, 7.44, 9.20] |
| $\mu_{T}$ | 1.89 | 1.81 | 1.80 | 1.83 |
| $\sigma_{T}$ | 0.21 | 0.21 | 0.22 | 0.25 |
| $c_{1}$ | 1.73 | 1.70 | 1.67 | 1.70 |
| $c_{2}$ | 1.97 | 1.91 | 1.88 | 1.95 |
| $c_{3}$ | 2.07 | 2.00 | 1.98 | 2.02 |
| $c_{4}$ | 2.20 | 2.15 | 2.12 | 2.17 |
| $c_{5}$ | 2.36 | 2.27 | 2.27 | 2.43 |
| $p$ | 0.50 | 0.53 | 0.47 | 0.52 |
|  |  |  |  |  |
| $G^{2}$ | 99.43 | 79.29 | 89.71 | 80.88 |

Table S6. Equal-variance signal (EVSD) detection model parameters and fits for each filler similarity condition for black males only.

|  | low | medium-low | medium-high | high |
| --- | --- | --- | --- | --- |
| $\mu_{T}$ | [1.68, 1.82, 1.97] | [1.53, 1.65, 1.79] | [1.54, 1.66, 1.79] | [1.54, 1.68, 1.82] |
| $\sigma_{T}$ | 1.00 | 1.00 | 1.00 | 1.00 |
| $c_{1}$ | 1.57 | 1.54 | 1.53 | 1.56 |
| $c_{2}$ | 2.11 | 2.04 | 1.97 | 2.06 |
| $c_{3}$ | 2.33 | 2.23 | 2.16 | 2.20 |
| $c_{4}$ | 2.61 | 2.53 | 2.44 | 2.49 |
| $c_{5}$ | 2.94 | 2.75 | 2.72 | 2.97 |
| $p$ | 0.50 | 0.53 | 0.47 | 0.53 |
|  |  |  |  |  |
| $G^{2}$ | 341.78 | 318.11 | 331.28 | 283.08 |

Table S7. Unequal-variance signal (UVSD) detection model parameters and fits for each filler similarity condition for black females only.

|  | low | medium-low | medium-high | high |
| --- | --- | --- | --- | --- |
| *d_a_* | [9.49, 11.40, 14.07] | [7.49, 9.00, 10.67] | [7.93, 9.74, 12.12] | [7.22, 8.63, 10.45] |
| $\mu_{T}$ | 1.93 | 1.81 | 1.98 | 1.84 |
| $\sigma_{T}$ | 0.17 | 0.20 | 0.21 | 0.21 |
| $c_{1}$ | 1.77 | 1.58 | 1.78 | 1.65 |
| $c_{2}$ | 2.03 | 1.92 | 2.10 | 1.93 |
| $c_{3}$ | 2.10 | 2.00 | 2.21 | 2.02 |
| $c_{4}$ | 2.24 | 2.13 | 2.35 | 2.23 |
| $c_{5}$ | 2.40 | 2.32 | 2.52 | 2.36 |
| $p$ | 0.49 | 0.48 | 0.56 | 0.52 |
|  |  |  |  |  |
| $G^{2}$ | 173.31 | 133.10 | 38.83 | 167.47 |

Table S8. Equal-variance signal (EVSD) detection model parameters and fits for each filler similarity condition for black females only.

|  | low | medium-low | medium-high | high |
| --- | --- | --- | --- | --- |
| $\mu_{T}$ | [1.80, 1.92, 2.07] | [1.68, 1.80, 1.92] | [1.87, 2.02, 2.18] | [1.67, 1.79, 1.91] |
| $\sigma_{T}$ | 1.00 | 1.00 | 1.00 | 1.00 |
| $c_{1}$ | 1.57 | 1.40 | 1.57 | 1.47 |
| $c_{2}$ | 2.25 | 2.10 | 2.40 | 2.08 |
| $c_{3}$ | 2.42 | 2.26 | 2.68 | 2.27 |
| $c_{4}$ | 2.76 | 2.52 | 2.99 | 2.69 |
| $c_{5}$ | 3.07 | 2.88 | 3.31 | 2.96 |
| $p$ | 0.49 | 0.48 | 0.56 | 0.52 |
|  |  |  |  |  |
| $G^{2}$ | 519.35 | 451.11 | 282.46 | 462.27 |

Table S9. Unequal-variance signal (UVSD) detection model parameters and fits for each filler similarity condition for white males only.

|  | low | medium-low | medium-high | high |
| --- | --- | --- | --- | --- |
| *d_a_* | [8.82, 10.87, 13.29] | [6.84, 8.34, 10.06] | [8.02, 9.66, 11.70] | [7.14, 8.74, 10.85] |
| $\mu_{T}$ | 2.07 | 1.85 | 1.81 | 1.86 |
| $\sigma_{T}$ | 0.19 | 0.22 | 0.19 | 0.22 |
| $c_{1}$ | 1.90 | 1.65 | 1.64 | 1.71 |
| $c_{2}$ | 2.16 | 1.93 | 1.88 | 1.94 |
| $c_{3}$ | 2.23 | 2.02 | 1.97 | 2.03 |
| $c_{4}$ | 2.37 | 2.19 | 2.13 | 2.17 |
| $c_{5}$ | 2.48 | 2.35 | 2.25 | 2.29 |
| $p$ | 0.53 | 0.47 | 0.45 | 0.48 |
|  |  |  |  |  |
| $G^{2}$ | 132.41 | 89.98 | 124.82 | 74.83 |

Table S10. Equal-variance signal (EVSD) detection model parameters and fits for each filler similarity condition for white males only.

|  | low | medium-low | medium-high | high |
| --- | --- | --- | --- | --- |
| $\mu_{T}$ | [1.94, 2.08, 2.23] | [1.68, 1.80, 1.93] | [1.64, 1.76, 1.88] | [1.63, 1.77, 1.91] |
| $\sigma_{T}$ | 1.00 | 1.00 | 1.00 | 1.00 |
| $c_{1}$ | 1.71 | 1.50 | 1.47 | 1.57 |
| $c_{2}$ | 2.41 | 2.06 | 2.00 | 2.06 |
| $c_{3}$ | 2.61 | 2.25 | 2.19 | 2.23 |
| $c_{4}$ | 2.96 | 2.59 | 2.52 | 2.52 |
| $c_{5}$ | 3.26 | 2.90 | 2.74 | 2.79 |
| $p$ | 0.53 | 0.47 | 0.45 | 0.48 |
|  |  |  |  |  |
| $G^{2}$ | 392.32 | 335.79 | 428.33 | 290.11 |

Table S11. Unequal-variance signal (UVSD) detection model parameters and fits for each filler similarity condition for white females only.

|  | low | medium-low | medium-high | high |
| --- | --- | --- | --- | --- |
| *d_a_* | [9.63, 11.92, 4.68] | [7.13, 8.60, 10.23] | [9.39, 11.37, 13.68] | [7.96, 9.69, 11.88] |
| $\mu_{T}$ | 1.96 | 1.88 | 1.89 | 1.94 |
| $\sigma_{T}$ | 0.16 | 0.22 | 0.17 | 0.20 |
| $c_{1}$ | 1.78 | 1.70 | 1.73 | 1.77 |
| $c_{2}$ | 2.06 | 2.02 | 2.02 | 2.05 |
| $c_{3}$ | 2.13 | 2.10 | 2.10 | 2.14 |
| $c_{4}$ | 2.22 | 2.26 | 2.22 | 2.26 |
| $c_{5}$ | 2.31 | 2.43 | 2.35 | 2.35 |
| $p$ | 0.47 | 0.52 | 0.53 | 0.48 |
|  |  |  |  |  |
| $G^{2}$ | 102.33 | 132.46 | 114.03 | 127.64 |

Table S12. Equal-variance signal (EVSD) detection model parameters and fits for each filler similarity condition for white females only.

|  | low | medium-low | medium-high | high |
| --- | --- | --- | --- | --- |
| $\mu_{T}$ | [1.88, 2.00, 2.16] | [1.70, 1.83, 1.96] | [1.74, 1.87, 2.00] | [1.81, 1.94, 2.08] |
| $\sigma_{T}$ | 1.00 | 1.00 | 1.00 | 1.00 |
| $c_{1}$ | 1.58 | 1.53 | 1.51 | 1.60 |
| $c_{2}$ | 2.33 | 2.23 | 2.28 | 2.26 |
| $c_{3}$ | 2.50 | 2.38 | 2.47 | 2.45 |
| $c_{4}$ | 2.72 | 2.71 | 2.72 | 2.73 |
| $c_{5}$ | 2.94 | 3.08 | 3.01 | 2.94 |
| $p$ | 0.47 | 0.52 | 0.53 | 0.48 |
|  |  |  |  |  |
| $G^{2}$ | 409.42 | 401.52 | 481.84 | 373.44 |

Table S13. pAUCs for black males only.

| condition 1 | condition 2 | *D*-value | *p*-value |
| --- | --- | --- | --- |
| low | medium-low | 1.18 | .118 |
| low | medium-high | -0.39 | .653 |
| low | high | -0.06 | .525 |
| medium-low | medium-high | -1.64 | .950 |
| medium-low | high | -1.26 | .897 |
| medium-high | high | 0.33 | .373 |
|  |  |  |  |

Table S14. pAUCs for black females only.

| condition 1 | condition 2 | *D*-value | *p*-value |
| --- | --- | --- | --- |
| low | medium-low | -0.48 | .685 |
| low | medium-high | 0.55 | .291 |
| low | high | -1.56 | .941 |
| medium-low | medium-high | 0.84 | .201 |
| medium-low | high | -0.85 | .803 |
| medium-high | high | -1.43 | .924 |
|  |  |  |  |

Table S15. pAUCs for white males only.

| condition 1 | condition 2 | *D*-value | *p*-value |
| --- | --- | --- | --- |
| low | medium-low | 0.29 | .387 |
| low | medium-high | -0.74 | .772 |
| low | high | -1.25 | .895 |
| medium-low | medium-high | -1.04 | .850 |
| medium-low | high | -1.56 | .941 |
| medium-high | high | -0.42 | .666 |
|  |  |  |  |

Table S16. pAUCs for white females only.

| condition 1 | condition 2 | *D*-value | *p*-value |
| --- | --- | --- | --- |
| low | medium-low | 0.64 | .262 |
| low | medium-high | 0.11 | .457 |
| low | high | -0.47 | .682 |
| medium-low | medium-high | -0.64 | .739 |
| medium-low | high | -1.62 | .947 |
| medium-high | high | -0.78 | .782 |
|  |  |  |  |

Table S17. Unequal-variance signal (UVSD) detection model parameters and fits for each filler similarity condition, allowing for unfair lineups, for black males only.

|  | low | medium-low | medium-high | high |
| --- | --- | --- | --- | --- |
| *d_a_* | [1.76, 2.05, 2.37] | [1.46, 1.77, 2.06] | [1.67, 2.01, 2.33] | [1.33, 1.69, 2.10] |
| $\mu_{T}$ | 2.57 | 2.36 | 2.31 | 2.19 |
| ${}_{T}$ | 1.16 | 1.48 | 1.26 | 1.26 |
| $\mu_{I}$ | 0.46 | 0.00 | 0.07 | 0.51 |
| ${}_{I}$ | 0.87 | 1.12 | 0.90 | 0.65 |
| $c_{Rej4}$ | 0.82 | 0.75 | 0.75 | 0.80 |
| $c_{Rej3}$ | 1.13 | 0.98 | 0.96 | 1.02 |
| $c_{Rej2}$ | 1.44 | 1.27 | 1.26 | 1.25 |
| $c_{Rej1}$ | 1.55 | 1.40 | 1.38 | 1.41 |
| $c_{ID1}$ | 1.68 | 1.62 | 1.58 | 1.61 |
| $c_{ID2}$ | 1.81 | 1.78 | 1.73 | 1.70 |
| $c_{ID3}$ | 1.98 | 1.92 | 1.90 | 1.93 |
| $c_{ID4}$ | 2.27 | 2.27 | 2.21 | 2.25 |
| $c_{ID5}$ | 2.66 | 2.65 | 2.59 | 2.50 |
|  |  |  |  |  |
| $p$ | 0.50 | 0.50 | 0.50 | 0.50 |
|  |  |  |  |  |
| $G^{2}$ | 5.49 | 20.88 | 14.86 | 17.97 |

Table S18. Unequal-variance signal (UVSD) detection model parameters and fits for each filler similarity condition, allowing for unfair lineups, for black females only.

|  | low | medium-low | medium-high | high |
| --- | --- | --- | --- | --- |
| *d_a_* | [1.83, 2.10, 2.38] | [2.03, 2.33, 2.65] | [2.17, 2.48, 2.82] | [1.72, 2.04, 2.39] |
| $\mu_{T}$ | 3.14 | 2.59 | 2.49 | 2.63 |
| ${}_{T}$ | 1.41 | 0.99 | 0.90 | 1.28 |
| $\mu_{I}$ | 0.61 | 0.55 | 0.00 | 0.55 |
| ${}_{I}$ | 0.96 | 0.74 | 1.06 | 0.66 |
| $c_{Rej4}$ | 0.97 | 0.92 | 0.72 | 0.89 |
| $c_{Rej3}$ | 1.25 | 1.05 | 1.09 | 1.08 |
| $c_{Rej2}$ | 1.47 | 1.29 | 1.33 | 1.33 |
| $c_{Rej1}$ | 1.60 | 1.40 | 1.48 | 1.41 |
| $c_{ID1}$ | 1.75 | 1.50 | 1.66 | 1.55 |
| $c_{ID2}$ | 1.86 | 1.62 | 1.75 | 1.65 |
| $c_{ID3}$ | 2.03 | 1.80 | 1.87 | 1.77 |
| $c_{ID4}$ | 2.42 | 2.07 | 2.09 | 2.18 |
| $c_{ID5}$ | 2.79 | 2.32 | 2.38 | 2.54 |
|  |  |  |  |  |
| $p$ | 0.50 | 0.50 | 0.50 | 0.50 |
|  |  |  |  |  |
| $G^{2}$ | 13.84 | 18.38 | 20.09 | 43.42 |

Table S19. Unequal-variance signal (UVSD) detection model parameters and fits for each filler similarity condition, allowing for unfair lineups, for white males only.

|  | low | medium-low | medium-high | high |
| --- | --- | --- | --- | --- |
| *d_a_* | [1.65, 1.93, 2.21] | [1.99, 2.25, 2.53] | [1.84, 2.18, 2.49] | [1.67, 2.04, 2.42] |
| $\mu_{T}$ | 3.08 | 2.44 | 2.69 | 2.37 |
| ${}_{T}$ | 1.49 | 1.11 | 1.34 | 1.17 |
| $\mu_{I}$ | 0.64 | 0.00 | 0.02 | 0.30 |
| ${}_{I}$ | 1.01 | 1.06 | 1.03 | 0.79 |
| $c_{Rej4}$ | 1.09 | 0.77 | 0.74 | 0.87 |
| $c_{Rej3}$ | 1.35 | 0.99 | 0.99 | 1.04 |
| $c_{Rej2}$ | 1.60 | 1.27 | 1.28 | 1.29 |
| $c_{Rej1}$ | 1.74 | 1.40 | 1.38 | 1.43 |
| $c_{ID1}$ | 1.91 | 1.56 | 1.53 | 1.62 |
| $c_{ID2}$ | 2.05 | 1.69 | 1.69 | 1.78 |
| $c_{ID3}$ | 2.20 | 1.84 | 1.82 | 1.93 |
| $c_{ID4}$ | 2.60 | 2.17 | 2.22 | 2.26 |
| $c_{ID5}$ | 3.01 | 2.47 | 2.62 | 2.58 |
|  |  |  |  |  |
| $p$ | 0.50 | 0.50 | 0.50 | 0.50 |
|  |  |  |  |  |
| $G^{2}$ | 18.92 | 26.49 | 25.58 | 15.68 |

Table S20. Unequal-variance signal (UVSD) detection model parameters and fits for each filler similarity condition, allowing for unfair lineups, for white females only.

|  | low | medium-low | medium-high | high |
| --- | --- | --- | --- | --- |
| *d_a_* | [2.07, 2.46, 2.86] | [1.85, 2.16, 2.46] | [1.84, 2.17, 2.50] | [1.85, 2.23, 2.65] |
| $\mu_{T}$ | 2.91 | 2.73 | 3.05 | 2.70 |
| ${}_{T}$ | 1.20 | 1.41 | 1.65 | 1.25 |
| $\mu_{I}$ | 0.40 | 0.00 | 0.08 | 0.50 |
| ${}_{I}$ | 0.77 | 1.03 | 0.94 | 0.64 |
| $c_{Rej4}$ | 1.00 | 0.85 | 0.82 | 0.93 |
| $c_{Rej3}$ | 1.16 | 1.07 | 1.04 | 1.13 |
| $c_{Rej2}$ | 1.35 | 1.33 | 1.33 | 1.37 |
| $c_{Rej1}$ | 1.45 | 1.46 | 1.45 | 1.52 |
| $c_{ID1}$ | 1.66 | 1.60 | 1.58 | 1.64 |
| $c_{ID2}$ | 1.83 | 1.70 | 1.71 | 1.80 |
| $c_{ID3}$ | 1.98 | 1.85 | 1.87 | 1.91 |
| $c_{ID4}$ | 2.23 | 2.17 | 2.16 | 2.18 |
| $c_{ID5}$ | 2.53 | 2.45 | 2.54 | 2.53 |
|  |  |  |  |  |
| $p$ | 0.50 | 0.50 | 0.50 | 0.50 |
|  |  |  |  |  |
| $G^{2}$ | 19.32 | 13.46 | 17.29 | 27.26 |

Table S21. Frequency counts of guilty suspect and innocent suspect identifications for each suspect-filler similarity condition at each level of confidence.

|  | low | |  | medium-low | |  | medium-high | |  | high | |
| --- | --- | --- | --- | --- | --- | --- | --- | --- | --- | --- | --- |
| Black males | TP | TA |  | TP | TA |  | TP | TA |  | TP | TA |
| 1 (0 – 50 %) | 36 | 13 |  | 36 | 11 |  | 37 | 11 |  | 46 | 8 |
| 2 (51 – 80 %) | 45 | 7 |  | 42 | 3 |  | 44 | 3 |  | 39 | 1 |
| 3 (81 – 100 %) | 137 | 1 |  | 131 | 3 |  | 125 | 0 |  | 128 | 1 |
| Total | 218 | 21 |  | 209 | 17 |  | 206 | 14 |  | 213 | 10 |
|  |  |  |  |  |  |  |  |  |  |  |  |
|  |  | |  |  | |  |  | |  |  | |
| Black females | TP | TA |  | TP | TA |  | TP | TA |  | TP | TA |
| 1 (0 – 50 %) | 39 | 22 |  | 35 | 17 |  | 30 | 5 |  | 34 | 14 |
| 2 (51 – 80 %) | 34 | 8 |  | 36 | 5 |  | 49 | 2 |  | 51 | 2 |
| 3 (81 – 100 %) | 188 | 4 |  | 176 | 3 |  | 162 | 4 |  | 175 | 1 |
| Total | 261 | 34 |  | 247 | 25 |  | 241 | 11 |  | 260 | 17 |
|  |  |  |  |  |  |  |  |  |  |  |  |
|  |  | |  |  | |  |  | |  |  | |
| White males | TP | TA |  | TP | TA |  | TP | TA |  | TP | TA |
| 1 (0 – 50 %) | 43 | 16 |  | 39 | 5 |  | 37 | 11 |  | 36 | 11 |
| 2 (51 – 80 %) | 46 | 8 |  | 42 | 6 |  | 42 | 6 |  | 42 | 0 |
| 3 (81 – 100 %) | 175 | 3 |  | 137 | 4 |  | 143 | 3 |  | 118 | 1 |
| Total | 264 | 27 |  | 218 | 15 |  | 222 | 20 |  | 196 | 12 |
|  |  |  |  |  |  |  |  |  |  |  |  |
|  |  |  |  |  |  |  |  |  |  |  |  |
| White females | TP | TA |  | TP | TA |  | TP | TA |  | TP | TA |
| 1 (0 – 50 %) | 29 | 11 |  | 33 | 11 |  | 27 | 10 |  | 32 | 9 |
| 2 (51 – 80 %) | 32 | 0 |  | 28 | 3 |  | 31 | 3 |  | 36 | 0 |
| 3 (81 – 100 %) | 170 | 2 |  | 189 | 2 |  | 208 | 1 |  | 158 | 0 |
| Total | 231 | 13 |  | 250 | 16 |  | 266 | 14 |  | 226 | 9 |
